# Modeling and detection of respiratory-related outbreak signatures

**DOI:** 10.1186/1472-6947-7-28

**Published:** 2007-10-05

**Authors:** Peter F Craigmile, Namhee Kim, Soledad A Fernandez, Bema K Bonsu

**Affiliations:** 1 Department of Statistics, 404 Cockins Hall, 1958 Neil Avenue, Columbus, OH 43210. USA; 2 Center for Biostatistics, M200 Starling Loving Hall, 320 West 10th Avenue, Columbus, OH 43210. USA; 3 Department of Pediatrics, Division of Emergency Medicine, Columbus Children's Hospital, 700 Children's Drive, Columbus, OH 43205. USA

## Abstract

**Background:**

Time series methods are commonly used to detect disease outbreak signatures (e.g., signals due to influenza outbreaks and anthrax attacks) from varying respiratory-related diagnostic or syndromic data sources. Typically this involves two components: (i) Using time series methods to model the baseline background distribution (the time series process that is assumed to contain no outbreak signatures), (ii) Detecting outbreak signatures using filter-based time series methods.

**Methods:**

We consider time series models for chest radiograph data obtained from Midwest children's emergency departments. These models incorporate available covariate information such as patient visit counts and smoothed ambient temperature series, as well as time series dependencies on daily and weekly seasonal scales. Respiratory-related outbreak signature detection is based on filtering the one-step-ahead prediction errors obtained from the time series models for the respiratory-complaint background.

**Results:**

Using simulation experiments based on a stochastic model for an anthrax attack, we illustrate the effect of the choice of filter and the statistical models upon radiograph-attributed outbreak signature detection.

**Conclusion:**

We demonstrate the importance of using seasonal autoregressive integrated average time series models (SARIMA) with covariates in the modeling of respiratory-related time series data. We find some homogeneity in the time series models for the respiratory-complaint backgrounds across the Midwest emergency departments studied. Our simulations show that the balance between specificity, sensitivity, and timeliness to detect an outbreak signature differs by the emergency department and the choice of filter. The linear and exponential filters provide a good balance.

## Background

Well-known, as well as previously uncharacterized infections continue to   (re)emerge around the globe. To avoid casualties from outbreaks of these   infections and from the potential criminal uses of bioagents, surveillance   systems are needed that have the capacity to identify such outbreaks   accurately and rapidly.  The accuracy and timeliness of biosurveillance   systems rests on the ability to model the uncertainty, severity, and   aberrancy of clinical symptoms that are likely to portend disease   outbreaks as expressed through the data monitoring system.  Shmueli [1] summarizes the problems that biosurveillance systems, in general, pose to   traditional statistical monitoring: (a) biosurveillance data may not be   independent or stationary; (b) non-traditional data are assumed to contain   earlier signature of an outbreak but this signal is weaker compared to   actual diagnosis data; (c) since there are no data that contain   bioterrorist outbreaks, outbreak patterns particularly as they would   manifest in non-traditional data streams are unknown; (d) biosurveillance   data are assumed to have no bioterrorist outbreaks, but the natural   outbreaks add up to the background noise. The key issue at hand is to   design statistical modeling and detection methods that can address these   problems.  

Among children, respiratory symptoms are an attractive target for surveillance. These are a prominent feature of many childhood epidemics and an early presentation of diseases like avian influenza, severe acute respiratory syndrome (SARS), and inhalational anthrax that have recently come to the public's attention. Unfortunately, respiratory complaints are also a feature of many common childhood illnesses, reducing the ability of biosurveillance systems to detect epidemics of greater public health concern. What is needed, therefore, is clinical information that is readily accessible and pre-processed in a manner that reflects the severity and aberrancy of respiratory symptoms. Using such data, discrimination between common childhood diseases and more serious respiratory epidemics would be possible.

Chest radiographs (X-rays), because they are readily available and are generally ordered by clinicians to evaluate respiratory complaints that are atypical or severe, have the potential to act as such a bio-monitoring and validation tool. In addition, detection based on models of radiograph ordering can indicate when in-depth follow-up is needed, as may occur when ordering of radiographs by clinicians is excessive for a given time of the year. Such in-depth review of radiographs may confirm clinical suspicions of an emerging epidemic or signal the need to perform a targeted review of medical charts to identify anomalous findings or groupings of aberrant findings that might herald the early stages of a respiratory-related outbreak.

In this article we consider time series methods for the modeling and detection of respiratory-related outbreak signatures based on chest radiograph ordering patterns from a number of pediatric emergency departments (EDs) located in the Midwestern region of the United States. These models include ambient temperature records collected in each city, as a covariate. We use the temperature series as a surrogate measure of the annual influenza season. Also, a patient visit count series is included in the models to account for variations between-EDs (like ED sizes) and within-EDs (day-of-week, for example). Addressing the fact that the underlying process is neither "independent or stationary", our interest is to model the underlying "respiratory-complaint background", using the available covariates and significant temporal dependencies present in the data. Without modeling the spatial dependence directly, we investigate whether or not there is evidence of spatial homogeneity in the statistical models across cities. We use filter-based prediction methods to indicate evidence of respiratory-related outbreaks (e.g., due to anthrax attack) using chest radiograph data. We describe the form and function of various filters that are commonly used to detect outbreak signatures. Using a stochastic model for an anthrax attack, we assess the performance of these methods. Since there are no data that contain outbreak patterns, the use of a model is key to providing realistic outbreak patterns that can accurately be used to evaluate these statistical detection methods.

Reis, Mandl, and others use time series methods to detect evidence of disease outbreaks at Boston Children's Hospital [2-4], modeling specific clinical complaints as deterministic trend and seasonalities plus a stationary autoregressive moving average (ARMA) process. They repeat this process for the visit counts instead of considering a joint modeling procedure. The detection algorithm filters one-step-ahead prediction errors [5], and looks for values in this residual process that exceed a predetermined threshold. Their simulations are based on less realistic deterministic outbreak models. Ivanov, et al. [6] use the Exponentially Weighted Moving Average smoother to measure timeliness, sensitivity, and specificity of free-text chief complaints (information describing patient's status on the ED visit). They indicate that the methods are good for detecting relatively large seasonal outbreaks, but not for small outbreaks. Burkom, et al. [7] extend this approach using Bayes Belief Networks to improve detection sensitivity and timeliness. There are also a number of wavelet-based and smoothing-based methods that can be used to monitor and detect abnormalities of unknown form, occurring over different time scales [1,8,9]. The use of scan statistics [10-12] has also gained popularity in recent years. Many of the methods based on scan statistics use fixed-effect models for biosurveillance (also see, e.g., [13] and [14] for other methods based on fixed-effect models).

Our manuscript is organized as follows: we start by summarizing the data of interest, and propose a statistical model for the ED data in each city. Using the growing literature on the subject, we outline a stochastic model for an anthrax outbreak along with a healthcare utilization model for simulating people entering the ED. We then describe the methodology and theory for detecting unexpected outbreak signatures in time series sources using filters. We examine the form and function of the filters used for detection. In the results section, we explore the time series models obtained for each Midwest ED included in our study. We assess these detection methods using a simulated anthrax attack, and we end with a discussion.

## Methods

### A chest radiograph model for respiratory-complaints

**Figure 1:**
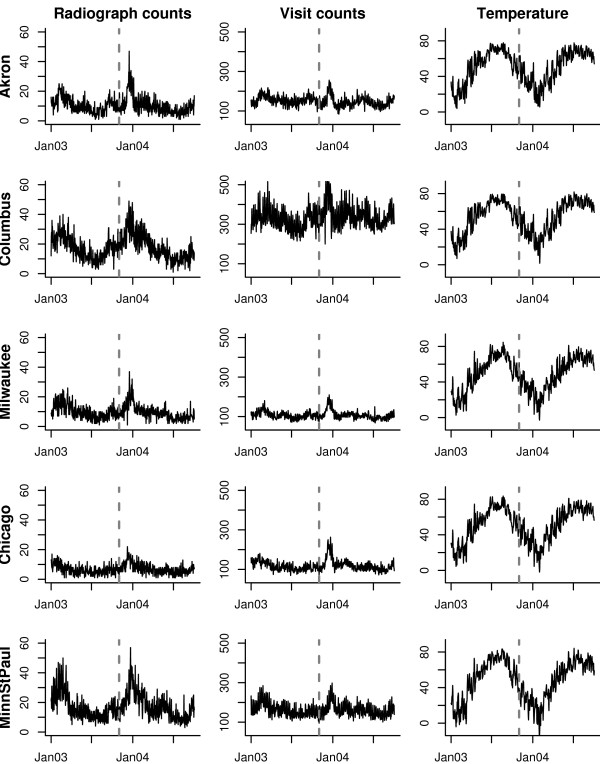
**Time series plots**. For each of the five metropolitan children's hospitals, plots of the daily chest radiograph counts (left), daily ED visit counts (middle) and daily temperature (right). The vertical dotted line indicates the separation between the training and test data.

The data of interest consist of daily counts of ED visits and chest radiographs taken between January 1st, 2003 and September 9th, 2004, in five metropolitan children's hospitals in the Midwest of the USA (Minneapolis/St. Paul, Milwaukee, Chicago, Akron, and Columbus), supplemented with time series of daily average temperature, obtained from the Average Daily Temperature Archive at The University of Dayton [15]. These series are shown in Figure 1. There seems to be strong seasonal component in the chest radiograph and visit series, for all the cities, which is negatively correlated with temperature. Although it could be argued that we do not have enough years of data to prove this empirically, it is expected that a higher number of respiratory complaints is associated with colder temperatures.

We now discuss two aspects of the statistical model for these daily chest radiograph counts: distribution and scale. Although the outcome variable of interest is counts, like other researchers in the field [1-4,6-9], we model the data using a Gaussian rather than Poisson process. The reasons are: (i) A normal approximation to the Poisson random variable is reasonable when the mean number of radiograph counts is large, as in this study; (ii) Gaussian time series models are easier to fit, diagnose, and interpret; (iii) The theory for filter-based methods commonly used for detecting the outbreak signature in such data is well developed for Gaussian processes. In some applications, count data are transformed when Gaussian models are used. Transformations, such as log or square root, are used to parameterize multiplicative models or to stabilize the variance. We use the original scale instead of transforming the data, like other researchers in the area, for ease of interpretation. This is especially important when we analyze filter-based detection methods in this article. For the same reason, we do not model the number of chest radiographs per daily ED visits (i.e., the proportion of radiographs). The filter-based theory, allows us to *directly *assess the effect of additive stochastic outbreaks signatures upon the radiograph series.

In our study, the first ten months of data (Jan to Oct, 2003) were used as the training set, while the remaining ten months (Nov, 2003 to Sep, 2004) were used, as a test dataset, to evaluate the model and detection methods.

Since the goal is to predict the number of chest radiographs, for each city we fit a linear time series regression model, using the number of chest radiographs as the response, and the number of visits and temperature as predictor variables. We smooth the temperature series, because we believe that long-range temporal trends are more predictive of chest radiograph counts. Let {*R*_*k*,*t*_} denote the number of chest radiographs for the ED in city *k *(*k *= 1, 2, ..., 5) on day *t *and let

*R*_*k*,*t *_= *β*_0,*k *_+ *β*_*V*,*k *_*V*_*k*,*t *_+ *β*_*T*,*k*_*T*_*k*,*t *_+ *X*_*k*,*t*_.

Here {*V*_*k*,*t*_} are the visit counts for city *k *and {*T*_*k*,*t*_} is the smoothed time series of temperatures for city *k*, filtered by taking a thirty day moving average to remove intra-month variation. To complete the model we assume that {*X*_*k*,*t*_} is a zero mean stationary time series (we discuss the consequences of this assumption in the Discussion), that we shall represent using a seasonal autoregressive integrated moving average process (SARIMA). The SARIMA model (defined in the Appendix) allows for simultaneous modeling of dependencies on both the day as well as the weekly seasonal scales. To aid in the comparison of the dependencies across cities, the order of the time series model, as determined by choosing the autoregressive (*p*_*k *_and *P*_*k*_) and moving average orders (*q*_*k *_and *Q*_*k*_), will be the same for each city *k*.

### A stochastic model for an anthrax outbreak

We now propose a simple stochastic model for an inhalational anthrax outbreak, based on the work of Buckeridge, et al. and Brookmeyer et al. [16,17]. As proposed by these authors, our model incorporates two elements:

1. A stochastic model of infection and progression of the disease.

2. A model of health-care-utilization that, on a day-by-day-basis, tracks the behavior of each infected individual.

Any inhalational anthrax outbreak starts with the *dispersion *of the anthrax spores. Once spores are inhaled by a subject, in the *incubation *stage spores either *germinate *or are *cleared *out the lung. For the spores that germinate during incubation, the later stages of the disease are the *prodromal *and *fulminant *stages, followed by *death*. Buckeridge, et al. [16] model the spread of spores over a grid covering the Norfolk, Virginia region using a Gaussian plume model. Their model considers the source and strength of the anthrax attack, along with prevalent wind directions.

Instead of using the region-based approach of Buckeridge, et al., we use the individual-based infection scheme of Brookmeyer, et al. [17]. Although it is known that anthrax spores can survive for long periods of time in the environment, we consider a small scale scenario. Since the population of interest is the individuals attending children's emergency departments, we assume an outbreak that affects a fixed number, *N *say, of children. Brookmeyer, et al. [17] define the infection probability of an individual exposed to inhalational anthrax using a competing risk model, modeling the dynamics of spore clearance and germination. Let *θ *represent the hazard rate per unit time (days, say) that a spore is cleared from the lung and *λ *be the rate of germination. Suppose that each individual inhales a dose of *D *spores. Then, the probability that at least one spore germinates is called the attack rate (AR) and is calculated using a Poisson approximation, as

*AR *= 1 - *e*^-*Dλ*/(*λ*+*θ*)^.

For a given attack rate, the probability that at least one of the *D *spores germinates within *t *days is given by

*F*(*t*) = 1 - (1 - *AR*)^(1-exp(-(*λ*+*θ*)*t*))^.

Note that the limit, lim_*t *→ ∞ _*F*(*t*) = AR. Based on a statistical analysis of an anthrax outbreak that occurred in Sverdlovsk, Russia, Brookmeyer, et al. [17] estimate hazard rates of between 0.05 and 0.11 for *θ *(which is compatible with *θ *= 0.07 obtained from animal studies for an AR of 0.5). The value of *λ *is not estimated in their data analysis – based on animal studies they found that the rate *λ *lies between 5 × 10^-7 ^and 10^-5^. Buckeridge, et al. [16] propose log normal models for the duration of the prodromal and fulminant stages, based on the 2001 anthrax attack in the United States. The median duration of the prodromal stage was 12.18 days, with a dispersion of 1.41, and the median duration of the fulminant stage was 1.5 days with a dispersion again of 1.41.

Next, we describe a simplified health-utilization model for people entering the ED, based on ideas discussed in [16]. During the incubation stage, we assume that no infected people enter the ED. Non-infected people that enter the ED for other chest problems are part of the background data. During the prodromal and fulminant stages we assume a simple Markov model of utilization: each infected subject is a Bernoulli event, independent across days. At the prodromal stage people enter the ED with probability *P*_*d *_on a weekday and *P*_*w *_on weekends. At the fulminant stage the probability of entering the ED, *P*_*f *_say, is larger than the probabilities in the prodromal stage. The reason is that at the fulminant stage, the anthrax symptoms are similar to those of a heart attack and therefore people enter the ED with higher probability. The differentiation between weekday and weekend is irrelevant at this stage. We suppose that a small percentage of people in the prodromal or fulminant stages are misdiagnosed and thus need to re-enter the system. Also, we assume that people can potentially be misdiagnosed a maximum of two times during the same attack (10% in the first visit and 5% in the second visit). The probabilities of entering the ED after being misdiagnosed are increased by an additive factor, *C*, for every additional entry. Our model allows for a small probability of drop-out, to account for other ways of leaving the system (e.g., pharmacy visit). The health-utilization model could easily be extended to include, e.g., varying probabilities of entering the ED by stage/time, and/or more advanced ways to exit the system.

### Filter-based outbreak signature detection

The main idea of filter-based methods is to create a detection process {*D*_*k*,*t*_}, for each time point *t*, which is a weighted average of the diagnostic or syndromic data to be used for the detection of an outbreak signature. The weights that appear in {*D*_*k*,*t*_} are defined using the form of the time series model and a filter, {*a*_*l*_} say. Extreme positive values of the detection process at a time point indicate a possible outbreak signature. The definition of the detection processes has its origin in process control [5], and is popularly used in the context of biosurveillance [1-4,6,8]. Naturally, the form of the filter has an important effect in the detection process.

A common parametric approach that we follow in this study is to first obtain the residual process by subtracting off the non-stationary part of the model (i.e., the effect of the covariates). We then define the filter {*c*_*k*,*j*_}, that first decorrelates (whitens) this residual process using the one-step-ahead prediction errors and second filters this whitened series using {*a*_*l*_} to yield the detection process {*D*_*k*,*t*_}. Commonly used examples of the filter {*a*_*l*_} include the differencing, moving average, or exponential filter. For a fixed value of *α*, let 1 - *α *denote the specificity (the probability of no detection, when there is no actual outbreak signature). We declare evidence of an outbreak signature at time *t *if the detection process at that time point, *D*_*k*,*t*_, exceeds a threshold *τ*_*k*,*α*_, calculated using the data or the process. Further details are given in the Appendix.

To understand the detection process {*D*_*k*,*t*_} for different choice of filters, {*a*_*l*_}, one should consider the filter involved in the calculation of {*D*_*k*,*t*_} using the residual series, {*X*_*k*,*t*_}. Using the results from the Appendix, at time point *t*,

Dk,t=∑h=0L−1ah∑j=0mck,jXk,t−h−j=∑l=0m+L−1fk,lXk,t−l.
 MathType@MTEF@5@5@+=feaafiart1ev1aaatCvAUfKttLearuWrP9MDH5MBPbIqV92AaeXatLxBI9gBaebbnrfifHhDYfgasaacH8akY=wiFfYdH8Gipec8Eeeu0xXdbba9frFj0=OqFfea0dXdd9vqai=hGuQ8kuc9pgc9s8qqaq=dirpe0xb9q8qiLsFr0=vr0=vr0dc8meaabaqaciaacaGaaeqabaqabeGadaaakeaacqWGebardaWgaaWcbaGaem4AaSMaeiilaWIaemiDaqhabeaakiabg2da9maaqahabaGaemyyae2aaSbaaSqaaiabdIgaObqabaaabaGaemiAaGMaeyypa0JaeGimaadabaGaemitaWKaeyOeI0IaeGymaedaniabggHiLdGcdaaeWbqaaiabdogaJnaaBaaaleaacqWGRbWAcqGGSaalcqWGQbGAaeqaaOGaemiwaG1aaSbaaSqaaiabdUgaRjabcYcaSiabdsha0jabgkHiTiabdIgaOjabgkHiTiabdQgaQbqabaaabaGaemOAaOMaeyypa0JaeGimaadabaGaemyBa0ganiabggHiLdGccqGH9aqpdaaeWbqaaiabdAgaMnaaBaaaleaacqWGRbWAcqGGSaalcqWGSbaBaeqaaOGaemiwaG1aaSbaaSqaaiabdUgaRjabcYcaSiabdsha0jabgkHiTiabdYgaSbqabaaabaGaemiBaWMaeyypa0JaeGimaadabaGaemyBa0Maey4kaSIaemitaWKaeyOeI0IaeGymaedaniabggHiLdGccqGGUaGlaaa@6D11@

In this expression, {*f*_*k*,*l*_} is the filter defined by the convolution of the filters {*a*_*l*_} and {*c*_*k*,*l*_}. Hence, we conclude that the detection of outbreaks not only depends on the choice of filter, but on the statistical properties of the time series model which defines {*c*_*k*,*l*_}. We will now focus on the effect of {*a*_*l*_} (we will investigate the effect of changing the time series model in the Simulations section). As Reis, et al did [2], we examine four different filters, {*a*_*l*_}, each of which is a form of difference filter. Each filter is an average of a number of days close to the time point minus a weighted average of the remaining values in the past:

1. 1-day filter: {*a*_*l*_} = (1, 0, 0, 0, 0, 0, 0);

2. 7-day filter: {*a*_*l*_} = (1, 1, 1, 1, 1, 1, 1)/7;

3. Linear filter: {*a*_*l*_} = (7, 6, 5, 4, 3, 2, 1)/28;

4. Exponential filter: {*a*_*l*_} = (64, 32, 16, 8, 4, 2, 1)/127.

**Figure 2:**
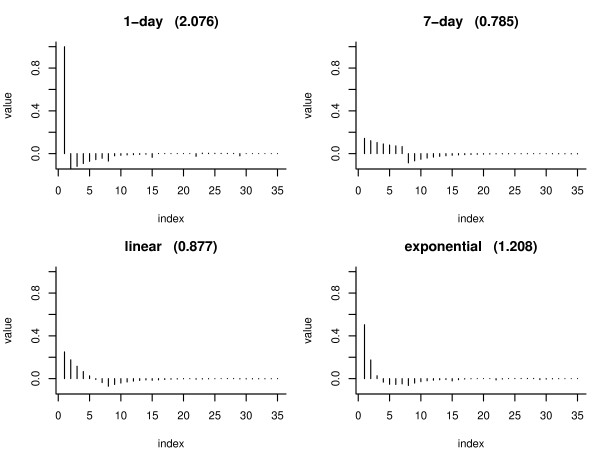
**Filter plots**. Plots of the four filters {*f*_*k*, *l*_} that can be used to calculate the detection process *D*_*k*, *t *_for the Columbus series. The number in parentheses after the filter name is *τ*_*k*, *α*_, calculated using a normal approximation.

The filters {*f*_*k*,*l*_} for the detection processes vary according to the amount of autocorrelation within each time series. As an illustration, Figure 2 displays the {*f*_*k*,*l*_} filter obtained when *m *= 28 for each of the four {*a*_*l*_} filters defined above, using a SARIMA model based on the Akron data (details of the SARIMA model are shown in the Results section).

1. When {*a*_*l*_} is a 1-day filter, the {*f*_*k*,*l*_} filter consists of the current time point of the process minus a smaller weighted decaying average of the past values. The weekly seasonal terms in the SARIMA model are reflected in this filter.

2. When {*a*_*l*_} is the 7-day filter, {*f*_*k*,*l*_} is a weighted average of the last 7 days (with most weight on the current day), minus a weighted decaying average of the remaining past days. The weekly seasonal terms are not as strong, compared to the filter that is the convolution of the 1-day filter.

3. When {*a*_*l*_} is the linear filter, {*f*_*k*,*l*_} is an average of 6 days in the past. Far more weight is put on the current day relative to the previous 6 days. From this we subtract an average of values previous to the 6 days. Most of the weight from the second average comes from around days 8–10 in the past.

4. When {*a*_*l*_} is the exponential filter, {*f*_*k*,*l*_} is a combination of the 4 most recent days (mostly the current day, very little by the fourth day). We subtract an average of the past values (mostly days 6 – 11 in the past).

In Figure 2, the numbers within the parentheses at the top of each panel show the value of the threshold *τ*_*k*,0.03_, calculated using the normal approximation used in the Appendix (equation 10), assuming an innovation variance of σk2
 MathType@MTEF@5@5@+=feaafiart1ev1aaatCvAUfKttLearuWrP9MDH5MBPbIqV92AaeXatLxBI9gBaebbnrfifHhDYfgasaacH8akY=wiFfYdH8Gipec8Eeeu0xXdbba9frFj0=OqFfea0dXdd9vqai=hGuQ8kuc9pgc9s8qqaq=dirpe0xb9q8qiLsFr0=vr0=vr0dc8meaabaqaciaacaGaaeqabaqabeGadaaakeaaiiGacqWFdpWCdaqhaaWcbaGaem4AaSgabaGaeGOmaidaaaaa@30F4@ = 1 for the process. We examine the effects upon the sensitivity, specificity, and timeliness to detect anthrax outbreaks in the simulations that we present later.

## Results

### Time series modeling

We fit the chest radiograph model given by (1) to the first ten months of data for each ED in city *k*. After specifying a model for {*R*_*k*,*t*_}, we have a regression model with time series errors, that can be fit using standard maximum likelihood methods. We selected the order of the SARIMA model for the time series errors, {*X*_*k*,*t*_}, as defined by (4) in the Appendix using standard identification techniques based on the sample autocorrelation and partial autocorrelation (e.g., [18]). To facilitate the comparison of the time series models across cities, we restricted to same order of model for each series. The orders *p*_*k *_= 1 and *q*_*k *_= 1, correspond to a single autoregressive and moving average term on the daily scale, equivalent to observing an autoregressive process of first order with measurement error ([19], Exercise 2.9). With a seasonal period of seven days (*s *= 7), we set *P*_*k *_= 1 and *Q*_*k *_= 1, so that the random seasonal component is a combination of an autoregressive and moving average term, each of first order over a period of seven days. The seasonal component of the time series model corresponds to an evolution of a first order autoregressive process with a measurement error over the weeks.

**Figure 3:**
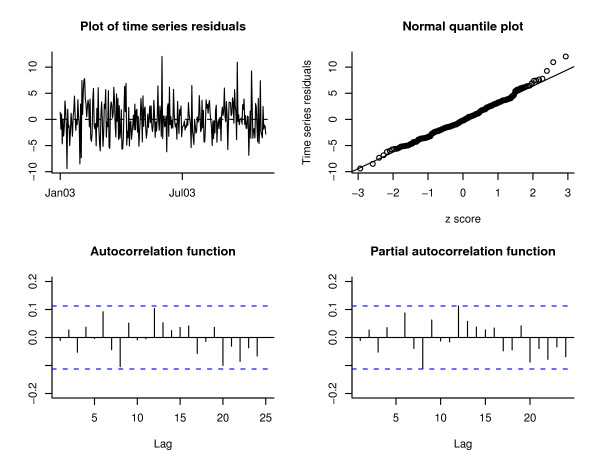
**Time series residual plots**. Summary diagnostic plots of the time series residuals based on the first ten months of training data from the Akron ED.

The model fit for the data in each city was assessed using diagnostic plots (time series plots, normal quantile plots, autocorrelation and partial autocorrelation plots, and the spectral estimates) of the estimated innovations of the time series component. Figure 3 shows some of these diagnostic plots for the Akron data (the residuals plots for the other cities were similar). Except for a few extremely positive values, the estimated time series looks stationary. The normal approximation is good, as evidenced by the straight line on the normal quantile plot. The plots of the sample autocorrelation and partial autocorrelations functions of the time series innovations lie inside the dotted confidence bounds for a white noise process (i.e., are samples of uncorrelated time series errors).

The parameter estimates for each city are summarized in Table 1. The standard errors for each parameter are shown in parentheses after each estimate. We note some homogeneities in the results across the EDs for each city. The intercept of the model, β^0,k
 MathType@MTEF@5@5@+=feaafiart1ev1aaatCvAUfKttLearuWrP9MDH5MBPbIqV92AaeXatLxBI9gBaebbnrfifHhDYfgasaacH8akY=wiFfYdH8Gipec8Eeeu0xXdbba9frFj0=OqFfea0dXdd9vqai=hGuQ8kuc9pgc9s8qqaq=dirpe0xb9q8qiLsFr0=vr0=vr0dc8meaabaqaciaacaGaaeqabaqabeGadaaakeaaiiGacuWFYoGygaqcamaaBaaaleaacqaIWaamcqGGSaalcqWGRbWAaeqaaaaa@31BD@, is positive for all cities, being largest in magnitude for Columbus, and smallest in magnitude for Chicago. The parameter estimate, β^V,k
 MathType@MTEF@5@5@+=feaafiart1ev1aaatCvAUfKttLearuWrP9MDH5MBPbIqV92AaeXatLxBI9gBaebbnrfifHhDYfgasaacH8akY=wiFfYdH8Gipec8Eeeu0xXdbba9frFj0=OqFfea0dXdd9vqai=hGuQ8kuc9pgc9s8qqaq=dirpe0xb9q8qiLsFr0=vr0=vr0dc8meaabaqaciaacaGaaeqabaqabeGadaaakeaaiiGacuWFYoGygaqcamaaBaaaleaacqWGwbGvcqGGSaalcqWGRbWAaeqaaaaa@3204@, relating visit counts and the radiograph counts is significantly different from zero, and positive, indicating, as one would expect, that a large number of ED visits are associated with a larger number of radiographs. The parameter estimate, β^T,k
 MathType@MTEF@5@5@+=feaafiart1ev1aaatCvAUfKttLearuWrP9MDH5MBPbIqV92AaeXatLxBI9gBaebbnrfifHhDYfgasaacH8akY=wiFfYdH8Gipec8Eeeu0xXdbba9frFj0=OqFfea0dXdd9vqai=hGuQ8kuc9pgc9s8qqaq=dirpe0xb9q8qiLsFr0=vr0=vr0dc8meaabaqaciaacaGaaeqabaqabeGadaaakeaaiiGacuWFYoGygaqcamaaBaaaleaacqWGubavcqGGSaalcqWGRbWAaeqaaaaa@3200@, relating the smooth temperature and radiograph counts is negative, indicating a significant negative association between these two quantities across all cities. The values of the autoregressive and moving average term on the daily scale (φ^k,1
 MathType@MTEF@5@5@+=feaafiart1ev1aaatCvAUfKttLearuWrP9MDH5MBPbIqV92AaeXatLxBI9gBaebbnrfifHhDYfgasaacH8akY=wiFfYdH8Gipec8Eeeu0xXdbba9frFj0=OqFfea0dXdd9vqai=hGuQ8kuc9pgc9s8qqaq=dirpe0xb9q8qiLsFr0=vr0=vr0dc8meaabaqaciaacaGaaeqabaqabeGadaaakeaaiiGacuWFgpGzgaqcamaaBaaaleaacqWGRbWAcqGGSaalcqaIXaqmaeqaaaaa@31D7@ and θ^k,1
 MathType@MTEF@5@5@+=feaafiart1ev1aaatCvAUfKttLearuWrP9MDH5MBPbIqV92AaeXatLxBI9gBaebbnrfifHhDYfgasaacH8akY=wiFfYdH8Gipec8Eeeu0xXdbba9frFj0=OqFfea0dXdd9vqai=hGuQ8kuc9pgc9s8qqaq=dirpe0xb9q8qiLsFr0=vr0=vr0dc8meaabaqaciaacaGaaeqabaqabeGadaaakeaaiiGacuWF4oqCgaqcamaaBaaaleaacqWGRbWAcqGGSaalcqaIXaqmaeqaaaaa@31D4@, respectively) are similar in value across all cities. Comparing these parameters, we can see that the strength of daily correlations is weakest for Milwaukee. Except for Chicago, the weekly seasonal autoregressive and moving average terms (Φ^k,1
 MathType@MTEF@5@5@+=feaafiart1ev1aaatCvAUfKttLearuWrP9MDH5MBPbIqV92AaeXatLxBI9gBaebbnrfifHhDYfgasaacH8akY=wiFfYdH8Gipec8Eeeu0xXdbba9frFj0=OqFfea0dXdd9vqai=hGuQ8kuc9pgc9s8qqaq=dirpe0xb9q8qiLsFr0=vr0=vr0dc8meaabaqaciaacaGaaeqabaqabeGadaaakeaacuqHMoGrgaqcamaaBaaaleaacqWGRbWAcqGGSaalcqaIXaqmaeqaaaaa@3191@ and Θ^k,1
 MathType@MTEF@5@5@+=feaafiart1ev1aaatCvAUfKttLearuWrP9MDH5MBPbIqV92AaeXatLxBI9gBaebbnrfifHhDYfgasaacH8akY=wiFfYdH8Gipec8Eeeu0xXdbba9frFj0=OqFfea0dXdd9vqai=hGuQ8kuc9pgc9s8qqaq=dirpe0xb9q8qiLsFr0=vr0=vr0dc8meaabaqaciaacaGaaeqabaqabeGadaaakeaacuqHyoqugaqcamaaBaaaleaacqWGRbWAcqGGSaalcqaIXaqmaeqaaaaa@318E@, respectively) are similar in value. By looking at the pointwise 95% confidence intervals for these two parameters, we conclude that there is no significant weekly variation in the Chicago series. As expected the values of σ^k2
 MathType@MTEF@5@5@+=feaafiart1ev1aaatCvAUfKttLearuWrP9MDH5MBPbIqV92AaeXatLxBI9gBaebbnrfifHhDYfgasaacH8akY=wiFfYdH8Gipec8Eeeu0xXdbba9frFj0=OqFfea0dXdd9vqai=hGuQ8kuc9pgc9s8qqaq=dirpe0xb9q8qiLsFr0=vr0=vr0dc8meaabaqaciaacaGaaeqabaqabeGadaaakeaaiiGacuWFdpWCgaqcamaaDaaaleaacqWGRbWAaeaacqaIYaGmaaaaaa@3104@, the estimated variance of the time series innovations, differ by city.

**Table 1:** Parameter estimates obtained from the chest radiograph model for each of the different cities studied. The standard errors are shown in parentheses

	Akron	Columbus	Milwaukee	Chicago	MinnStPaul
β^0,k MathType@MTEF@5@5@+=feaafiart1ev1aaatCvAUfKttLearuWrP9MDH5MBPbIqV92AaeXatLxBI9gBaebbnrfifHhDYfgasaacH8akY=wiFfYdH8Gipec8Eeeu0xXdbba9frFj0=OqFfea0dXdd9vqai=hGuQ8kuc9pgc9s8qqaq=dirpe0xb9q8qiLsFr0=vr0=vr0dc8meaabaqaciaacaGaaeqabaqabeGadaaakeaaiiGacuWFYoGygaqcamaaBaaaleaacqaIWaamcqGGSaalcqWGRbWAaeqaaaaa@31BD@	10.98 (2.51)	12.73 (1.71)	10.84 (2.14)	3.18 (1.68)	9.15 (2.86)
β^V,k MathType@MTEF@5@5@+=feaafiart1ev1aaatCvAUfKttLearuWrP9MDH5MBPbIqV92AaeXatLxBI9gBaebbnrfifHhDYfgasaacH8akY=wiFfYdH8Gipec8Eeeu0xXdbba9frFj0=OqFfea0dXdd9vqai=hGuQ8kuc9pgc9s8qqaq=dirpe0xb9q8qiLsFr0=vr0=vr0dc8meaabaqaciaacaGaaeqabaqabeGadaaakeaaiiGacuWFYoGygaqcamaaBaaaleaacqWGwbGvcqGGSaalcqWGRbWAaeqaaaaa@3204@	0.05 (0.01)	0.06 (0.00)	0.06 (0.01)	0.05 (0.01)	0.11 (0.01)
β^T,k MathType@MTEF@5@5@+=feaafiart1ev1aaatCvAUfKttLearuWrP9MDH5MBPbIqV92AaeXatLxBI9gBaebbnrfifHhDYfgasaacH8akY=wiFfYdH8Gipec8Eeeu0xXdbba9frFj0=OqFfea0dXdd9vqai=hGuQ8kuc9pgc9s8qqaq=dirpe0xb9q8qiLsFr0=vr0=vr0dc8meaabaqaciaacaGaaeqabaqabeGadaaakeaaiiGacuWFYoGygaqcamaaBaaaleaacqWGubavcqGGSaalcqWGRbWAaeqaaaaa@3200@	-0.15 (0.03)	-0.24 (0.02)	-0.14 (0.02)	-0.05 (0.02)	-0.22 (0.03)
φ^k,1 MathType@MTEF@5@5@+=feaafiart1ev1aaatCvAUfKttLearuWrP9MDH5MBPbIqV92AaeXatLxBI9gBaebbnrfifHhDYfgasaacH8akY=wiFfYdH8Gipec8Eeeu0xXdbba9frFj0=OqFfea0dXdd9vqai=hGuQ8kuc9pgc9s8qqaq=dirpe0xb9q8qiLsFr0=vr0=vr0dc8meaabaqaciaacaGaaeqabaqabeGadaaakeaaiiGacuWFgpGzgaqcamaaBaaaleaacqWGRbWAcqGGSaalcqaIXaqmaeqaaaaa@31D7@	0.91 (0.05)	0.92 (0.07)	0.79 (0.13)	0.96 (0.05)	0.95 (0.03)
θ^k,1 MathType@MTEF@5@5@+=feaafiart1ev1aaatCvAUfKttLearuWrP9MDH5MBPbIqV92AaeXatLxBI9gBaebbnrfifHhDYfgasaacH8akY=wiFfYdH8Gipec8Eeeu0xXdbba9frFj0=OqFfea0dXdd9vqai=hGuQ8kuc9pgc9s8qqaq=dirpe0xb9q8qiLsFr0=vr0=vr0dc8meaabaqaciaacaGaaeqabaqabeGadaaakeaaiiGacuWF4oqCgaqcamaaBaaaleaacqWGRbWAcqGGSaalcqaIXaqmaeqaaaaa@31D4@	-0.74 (0.07)	-0.83 (0.11)	-0.69 (0.14)	-0.90 (0.07)	-0.89 (0.05)
Φ^k,1 MathType@MTEF@5@5@+=feaafiart1ev1aaatCvAUfKttLearuWrP9MDH5MBPbIqV92AaeXatLxBI9gBaebbnrfifHhDYfgasaacH8akY=wiFfYdH8Gipec8Eeeu0xXdbba9frFj0=OqFfea0dXdd9vqai=hGuQ8kuc9pgc9s8qqaq=dirpe0xb9q8qiLsFr0=vr0=vr0dc8meaabaqaciaacaGaaeqabaqabeGadaaakeaacuqHMoGrgaqcamaaBaaaleaacqWGRbWAcqGGSaalcqaIXaqmaeqaaaaa@3191@	0.88 (0.16)	0.88 (0.04)	0.83 (0.25)	-0.49 (0.34)	0.84 (0.08)
Θ^k,1 MathType@MTEF@5@5@+=feaafiart1ev1aaatCvAUfKttLearuWrP9MDH5MBPbIqV92AaeXatLxBI9gBaebbnrfifHhDYfgasaacH8akY=wiFfYdH8Gipec8Eeeu0xXdbba9frFj0=OqFfea0dXdd9vqai=hGuQ8kuc9pgc9s8qqaq=dirpe0xb9q8qiLsFr0=vr0=vr0dc8meaabaqaciaacaGaaeqabaqabeGadaaakeaacuqHyoqugaqcamaaBaaaleaacqWGRbWAcqGGSaalcqaIXaqmaeqaaaaa@318E@	-0.84 (0.17)	-0.99 (0.06)	-0.77 (0.28)	0.42 (0.36)	-0.73 (0.10)
σ^k2 MathType@MTEF@5@5@+=feaafiart1ev1aaatCvAUfKttLearuWrP9MDH5MBPbIqV92AaeXatLxBI9gBaebbnrfifHhDYfgasaacH8akY=wiFfYdH8Gipec8Eeeu0xXdbba9frFj0=OqFfea0dXdd9vqai=hGuQ8kuc9pgc9s8qqaq=dirpe0xb9q8qiLsFr0=vr0=vr0dc8meaabaqaciaacaGaaeqabaqabeGadaaakeaaiiGacuWFdpWCgaqcamaaDaaaleaacqWGRbWAaeaacqaIYaGmaaaaaa@3104@	10.71	18.09	12.45	6.46	18.33

### Simulations

In the simulations described in this section we used the following experimental design. For each city, we fit the regression time series model using the first ten months of data (training data). We used the second half of the data from November 1st, 2003 onwards and added outbreak-related counts to test for the detection of an outbreak signature.

**Figure 4:**
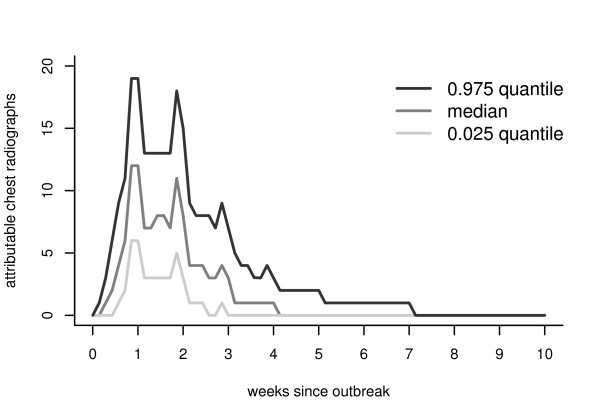
**Quantiles of the chest radiograph distribution attributable to an anthrax outbreak**. Plots of the day-by-day quantiles of the radiograph counts attributable to an anthrax attack, based on 500 simulated anthrax outbreaks.

We simulated an outbreak, as previously described, 500 times on 500 individuals using an attack rate of 0.5. The first day of the outbreak was randomly picked from a uniform distribution in the period December 5th, 2003 to June 22nd, 2004. (Making the earliest date later than November 1st, 2003, guarantees enough data for the filter-based methods to work with a prediction window of *m *= 28 days). In the absence of other information (e.g., Buckeridge, et al. [16] do not report their probabilities) we set the ED utilization probabilities to be: *P*_*d *_= 0.25 (weekday entry probability at the prodromal stage); *P*_*w *_= 0.4 (weekend entry probability at the prodromal stage) and *P*_*f *_= 0.80 (entry probability at the fulminant stage). The daily drop-out probability was set at 0.05. We assumed that with probability 0.9, the infected persons that enter the ED for the first time during a given outbreak receive a chest radiograph, i.e., 10% of the infected persons are misdiagnosed at the first visit. In a subsequent visit, 5% of the infected persons that re-enter the ED are misdiagnosed. The misdiagnosis additive factor, *C*, was assumed to be 0.05. We added the counts from the ED visits and subsequent number of chest radiographs generated from the healthcare utilization model on each day during the outbreak period. Let {*O*_*k*,*t*_} denote the number of chest radiographs attributable to the anthrax attack on day *t*. Since each simulated outbreak will be different, we start by summarizing the distribution of {*O*_*k*,*t*_}. Figure 4 shows a plot of the 0.025, 0.5 (i.e., median), and 0.975 quantiles calculated over the 500 simulated realizations for each day *t*. Examining the progression of the quantiles over time allows us to explore the center and tails of the extra radiograph count distribution. The counts increase rapidly in the first week, then stay fairly constant (except for the spikes), for a week and then slowly drop to zero after the second week. The small spikes are due to the different probabilities of entry in the prodromal stage. Although the shapes at each quantile are similar, the magnitude and duration of the extra radiographs counts differ. The counts drop to zero at different time points for the different quantiles. They drop to zero after 3 weeks for the 0.025 quantile, after 4 weeks for the 0.5 quantile, and after 7 weeks for the 0.975 quantile. These patterns in the observed time-varying distribution of the outbreak signature are a strong motivation to not use deterministic outbreak patterns, as used by some authors, since deterministic outbreak patterns do not give a realistic assessment of detection methods.

We declare that there is evidence of an outbreak signature in the radiograph data at time *t *if the outbreak process *D*_*k*,*t*_, defined by (2), exceeds a given threshold, *τ*_*k*,*α*_. Suppose that {*Y*_*k*,*t*_} is the process defined as the sum of {*O*_*k*,*t*_}, the extra radiograph counts attributable to the outbreak, and the radiograph process {*R*_*k*,*t*_} that follows model (1). Removing the non-stationary part due to the estimated effect of the covariates yields

Wk,t≡Yk,t−(β^0,k+β^V,kVk,t+β^T,kTk,t)≈Ok,t+Xk,t.
 MathType@MTEF@5@5@+=feaafiart1ev1aaatCvAUfKttLearuWrP9MDH5MBPbIqV92AaeXatLxBI9gBaebbnrfifHhDYfgasaacH8akY=wiFfYdH8Gipec8Eeeu0xXdbba9frFj0=OqFfea0dXdd9vqai=hGuQ8kuc9pgc9s8qqaq=dirpe0xb9q8qiLsFr0=vr0=vr0dc8meaabaqaciaacaGaaeqabaqabeGadaaakeaacqWGxbWvdaWgaaWcbaGaem4AaSMaeiilaWIaemiDaqhabeaakiabggMi6kabdMfaznaaBaaaleaacqWGRbWAcqGGSaalcqWG0baDaeqaaOGaeyOeI0IaeiikaGccciGaf8NSdiMbaKaadaWgaaWcbaGaeGimaaJaeiilaWIaem4AaSgabeaakiabgUcaRiqb=j7aIzaajaWaaSbaaSqaaiabdAfawjabcYcaSiabdUgaRbqabaGccqWGwbGvdaWgaaWcbaGaem4AaSMaeiilaWIaemiDaqhabeaakiabgUcaRiqb=j7aIzaajaWaaSbaaSqaaiabdsfaujabcYcaSiabdUgaRbqabaGccqWGubavdaWgaaWcbaGaem4AaSMaeiilaWIaemiDaqhabeaakiabcMcaPiabgIKi7kabd+eapnaaBaaaleaacqWGRbWAcqGGSaalcqWG0baDaeqaaOGaey4kaSIaemiwaG1aaSbaaSqaaiabdUgaRjabcYcaSiabdsha0bqabaGccqGGUaGlaaa@64AE@

By applying the filter to this series we obtain the detection process that we would observe in the presence of extra counts attributable to an outbreak:

∑l=0m+L−1fk,lWk,t−l≈∑l=0m+L−1fk,lOk,t−l+∑l=0m+L−1fk,lXk,t−l.
 MathType@MTEF@5@5@+=feaafiart1ev1aaatCvAUfKttLearuWrP9MDH5MBPbIqV92AaeXatLxBI9gBaebbnrfifHhDYfgasaacH8akY=wiFfYdH8Gipec8Eeeu0xXdbba9frFj0=OqFfea0dXdd9vqai=hGuQ8kuc9pgc9s8qqaq=dirpe0xb9q8qiLsFr0=vr0=vr0dc8meaabaqaciaacaGaaeqabaqabeGadaaakeaadaaeWbqaaiabdAgaMnaaBaaaleaacqWGRbWAcqGGSaalcqWGSbaBaeqaaOGaem4vaC1aaSbaaSqaaiabdUgaRjabcYcaSiabdsha0jabgkHiTiabdYgaSbqabaaabaGaemiBaWMaeyypa0JaeGimaadabaGaemyBa0Maey4kaSIaemitaWKaeyOeI0IaeGymaedaniabggHiLdGccqGHijYUdaaeWbqaaiabdAgaMnaaBaaaleaacqWGRbWAcqGGSaalcqWGSbaBaeqaaOGaem4ta80aaSbaaSqaaiabdUgaRjabcYcaSiabdsha0jabgkHiTiabdYgaSbqabaaabaGaemiBaWMaeyypa0JaeGimaadabaGaemyBa0Maey4kaSIaemitaWKaeyOeI0IaeGymaedaniabggHiLdGccqGHRaWkdaaeWbqaaiabdAgaMnaaBaaaleaacqWGRbWAcqGGSaalcqWGSbaBaeqaaOGaemiwaG1aaSbaaSqaaiabdUgaRjabcYcaSiabdsha0jabgkHiTiabdYgaSbqabaaabaGaemiBaWMaeyypa0JaeGimaadabaGaemyBa0Maey4kaSIaemitaWKaeyOeI0IaeGymaedaniabggHiLdGccqGGUaGlaaa@7644@

To examine the effect of the filter upon the outbreak signature we examine the standardized quantity

gk,t=∑l=0m+L−1fk,lOk,t−l/τk,α
 MathType@MTEF@5@5@+=feaafiart1ev1aaatCvAUfKttLearuWrP9MDH5MBPbIqV92AaeXatLxBI9gBaebbnrfifHhDYfgasaacH8akY=wiFfYdH8Gipec8Eeeu0xXdbba9frFj0=OqFfea0dXdd9vqai=hGuQ8kuc9pgc9s8qqaq=dirpe0xb9q8qiLsFr0=vr0=vr0dc8meaabaqaciaacaGaaeqabaqabeGadaaakeaacqWGNbWzdaWgaaWcbaGaem4AaSMaeiilaWIaemiDaqhabeaakiabg2da9maaqahabaGaemOzay2aaSbaaSqaaiabdUgaRjabcYcaSiabdYgaSbqabaGccqWGpbWtdaWgaaWcbaGaem4AaSMaeiilaWIaemiDaqNaeyOeI0IaemiBaWgabeaakiabc+caVGGaciab=r8a0naaBaaaleaacqWGRbWAcqGGSaalcqWFXoqyaeqaaaqaaiabdYgaSjabg2da9iabicdaWaqaaiabd2gaTjabgUcaRiabdYeamjabgkHiTiabigdaXaqdcqGHris5aaaa@50FB@

for the filters that we defined previously. We set *α *= 0.03, which corresponds to a false alarm rate of one per month [2]. Then, as shown in the Appendix, the threshold, *τ*_*k*,*α*_, is chosen by solving *P*(*D*_*k*,*t *_> *τ*_*k*,*α*_) = *α *for *τ*_*k*,*α*_. Either we can estimate this value from the data using the 1 - *α *quantile of the {*D*_*k*,*t*_} process of non-outbreak-based training data, or via a normal approximation, given by (10). There were some differences between the values of *τ*_*k*,*α *_for the two methods in the simulations we studied. We chose the normal approximation method as it tended to preserve the specificity across the filters and EDs that we considered. Scaling *g*_*k*,*t *_by *τ*_*k*,0.03 _and using the same simulated radiograph realizations due to the outbreak, allow us to compare the filtered signals consistently across filters.

**Figure 5:**
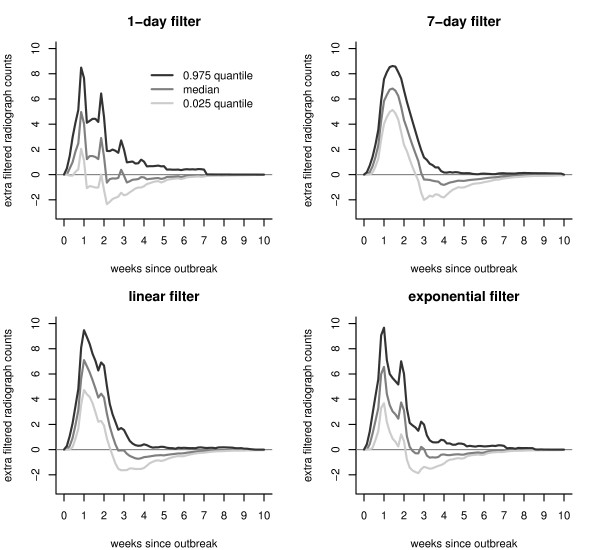
**Quantiles of the filtered chest radiograph distribution attributable to an anthrax outbreak**. Plots of the day-by-day quantiles of the filtered radiograph counts attributable to an anthrax attack, based on 500 simulated anthrax outbreaks. The filtering operation, as defined by (3), is based on the first ten months of training data from Akron, in combination with one of four filters (1-day, 7-day, linear and exponential). Each filtered signal is scaled by *τ*_*k*, 0.03_, calculated using a normal approximation, for comparisons to be made across filters.

The filtered series will be different for each simulated outbreak pattern realization. Just as in Figure 4 with the radiograph series, we calculate the 0.025, 0.5 (median), and 0.975 quantiles of the filtered radiograph series on each day *t*, based on the model fit to the Akron ED series. Figure 5 shows the time-varying quantiles for each of the four filters. Relative to the no-outbreak situation, positive values denote those time points for which there would be a greater chance of setting off a detection. Negative values denote the time periods that would actually decrease the chance of a detection. Except for the 0.975 quantile of the 1-day filter, each quantile of the filtered signal drops below zero for a period, and stabilizes at zero after that.

There are some similarities in the shapes of the three quantiles presented in Figures 4 and 5. Namely, the filtered signals increase over the first week, and then slowly decrease. The spikes in the distributions of the original signal are preserved for the 1-day filter, smoothed out for the 7-day filter, and partial smoothed over for the linear and exponential filters. Smoothing out the spikes tends to lead more sustained peaks (i.e., wider periods of higher sensitivity). The power of detection is smaller for the 1-day and 7-day than the linear and the exponential (as witnessed by smaller maximum heights in each of these stwo curves). For all filters, a drop below zero occurs immediately after the peaks; the counts go slowly back to zero. Periods for which the filtered signals are below zero inhibit detection and thus can mislead the prediction of the end of the outbreak signature.

**Figure 6:**
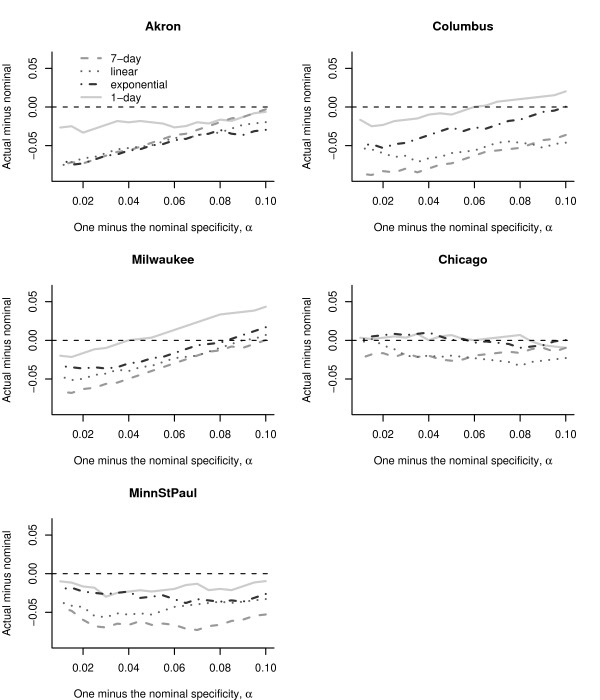
**The biases in the specificity**. A plot of the actual specificity (the specificity calculated using the training data) minus the nominal specificity (1 - *α*) for different values of *α*. The value of the detection threshold *τ*_*k*,*α*_, was calculated using a normal approximation.

**Figure 7:**
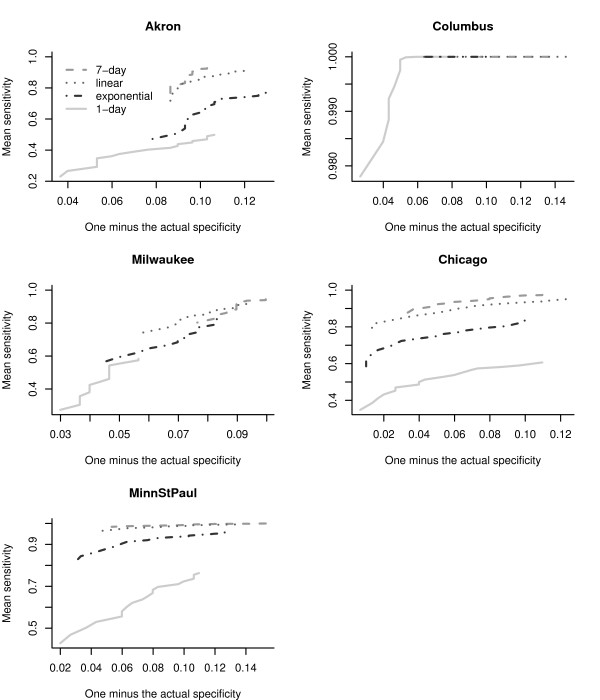
**Receiver operating characteristic curves**. Receiver operating characteristic curves (averaged over 500 simulations of an anthrax outbreak) for the detection of the anthrax outbreak signature in the chest radiographs at each of the five metropolitan children's hospitals.

We calculated the proportion of true non-detects in the absence of an outbreak signature, based on the test data (the specificity), as well as the proportion of true detects during the outbreak (the sensitivity) averaging over the 500 simulated anthrax outbreaks, for each filter and city. We calculated the actual specificity and sensitivity using the test data, for values of *α*, in the range 0.01 to 0.10 in steps of 0.005. Figure 6 displays the difference of actual specificity (calculated using the training data) and the nominal specificity (predetermined 1 - *α*) for different values of *α*. Curves above or below the horizontal dotted line at zero, indicate departures from the nominal *α*. The Chicago series preserves the nominal value of the specificity (curves are clustered around the zero line). For the Minneapolis-St.Paul series the actual specificity is biased downwards, and for the other cities, the departure from the nominal value changes as *α *increases. The choice of filter affected the calibration. To understand the tradeoff between the specificity and the  sensitivity we show the receiver operator characteristic (ROC) curves,  for each city, in Figure 7. For all cities, the 1-day filter has the poorest sensitivity. Except for Milwaukee, the 7-day filter tends to have higher sensitivities compared to the other filters, but also tends to have the poorest specificity. The exponential filter balances the specificity-sensitivity tradeoff.

Tables 2 and 3 display, respectively, the median and maximum detection times for each filter and city for our model. The 1-day filter performed poorly. The other filters are more comparable. We observed (results not shown) that the time to detection increases if the attack rate is reduced and also if the probabilities of entering the ED is delayed.

**Table 2:** Median time to detect an outbreak for each city and filter

	1-day	7-day	linear	exponential
Akron	3	1	1	2
Columbus	1	1	1	1
Milwaukee	4	1	1	1
Chicago	2	1	1	1
Minn/St. Paul	2	1	1	1

**Table 3:** Maximum time to detect an outbreak for each city and filter

	1-day	7-day	linear	exponential
Akron	9	9	7	7
Columbus	2	1	1	1
Milwaukee	9	7	7	7
Chicago	7	4	4	5
Minn/St. Paul	6	2	4	5

We now compare the performance of our model, as defined by equation (1), with three other models, in order to investigate the effect of different covariates and time series components upon outbreak signature detection. Remember {*R*_*k*,*t*_} are the number of chest radiographs for the ED in city *k *on day *t*, {*V*_*k*,*t*_} are the ED visit counts, and {*T*_*k*,*t*_} are the thirty day smoothed time series of temperature. For each day, *t*, and day of the week, *d *= 1, ..., 7, let *D*_*d*,*t *_be an indicator function that is one if that day is the *d*th day of the week, and zero otherwise. The four models we compare are:

1. Our covariates plus SARIMA errors model: *R*_*k*,*t *_= *β*_0,*k *_+ *β*_*V*,*k*_*V*_*k*,*t *_+ *β*_*T*,*k*_*T*_*k*,*t *_+ *X*_*k*,*t*_, where {*X*_*k*,*t*_} is the SARIMA model used in the Results section.

2. Covariates (with seasonality) with autoregressive moving average (ARMA) errors model: Rk,t=β0,k+βV,tVk,t+βT,kTk,t+∑d=16βD,d,kDd,t+Xk,t
 MathType@MTEF@5@5@+=feaafiart1ev1aaatCvAUfKttLearuWrP9MDH5MBPbIqV92AaeXatLxBI9gBaebbnrfifHhDYfgasaacH8akY=wiFfYdH8Gipec8Eeeu0xXdbba9frFj0=OqFfea0dXdd9vqai=hGuQ8kuc9pgc9s8qqaq=dirpe0xb9q8qiLsFr0=vr0=vr0dc8meaabaqaciaacaGaaeqabaqabeGadaaakeaacqWGsbGudaWgaaWcbaGaem4AaSMaeiilaWIaemiDaqhabeaakiabg2da9GGaciab=j7aInaaBaaaleaacqaIWaamcqGGSaalcqWGRbWAaeqaaOGaey4kaSIae8NSdi2aaSbaaSqaaiabdAfawjabcYcaSiabdsha0bqabaGccqWGwbGvdaWgaaWcbaGaem4AaSMaeiilaWIaemiDaqhabeaakiabgUcaRiab=j7aInaaBaaaleaacqWGubavcqGGSaalcqWGRbWAaeqaaOGaemivaq1aaSbaaSqaaiabdUgaRjabcYcaSiabdsha0bqabaGccqGHRaWkdaaeWaqaaiab=j7aInaaBaaaleaacqWGebarcqGGSaalcqWGKbazcqGGSaalcqWGRbWAaeqaaOGaemiraq0aaSbaaSqaaiabdsgaKjabcYcaSiabdsha0bqabaaabaGaemizaqMaeyypa0JaeGymaedabaGaeGOnaydaniabggHiLdGccqGHRaWkcqWGybawdaWgaaWcbaGaem4AaSMaeiilaWIaemiDaqhabeaaaaa@67AD@, where {*X*_*k*,*t*_} is an ARMA model (equation (4), without the Φ_*k *_and Θ_*k *_terms) with orders *p*_*k *_= 1 and *q*_*k *_= 1. Instead of modeling the weekly effect using a random seasonal component we use day-of-the week as a fixed effect (covariate).

3. Covariates and no time series errors: Rk,t=β0,k+βV,tVk,t+βT,kTk,t+∑d=16βD,d,kDd,t+Xk,t
 MathType@MTEF@5@5@+=feaafiart1ev1aaatCvAUfKttLearuWrP9MDH5MBPbIqV92AaeXatLxBI9gBaebbnrfifHhDYfgasaacH8akY=wiFfYdH8Gipec8Eeeu0xXdbba9frFj0=OqFfea0dXdd9vqai=hGuQ8kuc9pgc9s8qqaq=dirpe0xb9q8qiLsFr0=vr0=vr0dc8meaabaqaciaacaGaaeqabaqabeGadaaakeaacqWGsbGudaWgaaWcbaGaem4AaSMaeiilaWIaemiDaqhabeaakiabg2da9GGaciab=j7aInaaBaaaleaacqaIWaamcqGGSaalcqWGRbWAaeqaaOGaey4kaSIae8NSdi2aaSbaaSqaaiabdAfawjabcYcaSiabdsha0bqabaGccqWGwbGvdaWgaaWcbaGaem4AaSMaeiilaWIaemiDaqhabeaakiabgUcaRiab=j7aInaaBaaaleaacqWGubavcqGGSaalcqWGRbWAaeqaaOGaemivaq1aaSbaaSqaaiabdUgaRjabcYcaSiabdsha0bqabaGccqGHRaWkdaaeWaqaaiab=j7aInaaBaaaleaacqWGebarcqGGSaalcqWGKbazcqGGSaalcqWGRbWAaeqaaOGaemiraq0aaSbaaSqaaiabdsgaKjabcYcaSiabdsha0bqabaaabaGaemizaqMaeyypa0JaeGymaedabaGaeGOnaydaniabggHiLdGccqGHRaWkcqWGybawdaWgaaWcbaGaem4AaSMaeiilaWIaemiDaqhabeaaaaa@67AD@, where {*X*_*k*,*t*_} is a mean zero white noise process with innovation variance σk2
 MathType@MTEF@5@5@+=feaafiart1ev1aaatCvAUfKttLearuWrP9MDH5MBPbIqV92AaeXatLxBI9gBaebbnrfifHhDYfgasaacH8akY=wiFfYdH8Gipec8Eeeu0xXdbba9frFj0=OqFfea0dXdd9vqai=hGuQ8kuc9pgc9s8qqaq=dirpe0xb9q8qiLsFr0=vr0=vr0dc8meaabaqaciaacaGaaeqabaqabeGadaaakeaaiiGacqWFdpWCdaqhaaWcbaGaem4AaSgabaGaeGOmaidaaaaa@30F4@.

4. No covariates and ARMA errors: *R*_*k*,*t *_= *β*_0,*k *_+ *X*_*k*,*t*_, where {*X*_*k*,*t*_} is the same ARMA model as in Model 2.

**Figure 8:**
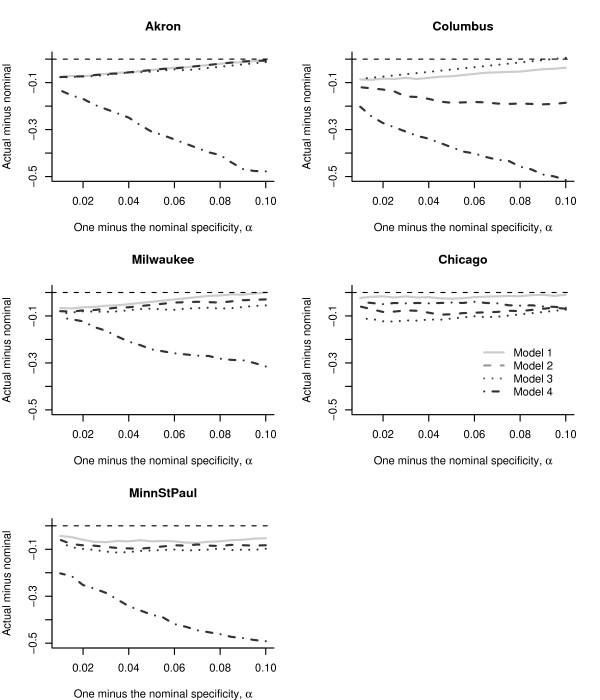
**The biases in the specificity for the four models**. A plot of the actual specificity (the specificity calculated using the training data) minus the nominal specificity (1 - *α*) for different values of *α *using a linear filter, for four different models. The value of the detection threshold *τ*_*k*,*α*_, was calculated using a normal approximation.

Figure 8 displays the difference of actual specificity (calculated using the training data) and the nominal specificity (the predetermined 1 - *α*) for different values of *α *for these four models. For illustration we use the linear filter, which represents a compromise between the actual specificity and sensitivity (Across all four models, the 1-day filter always had specificities closest to nominal and the 7-day filter had specificities furthest away). Except for Columbus, Model 1 achieved a specificity closer to the nominal value across all EDs (Model 3 outperforms Model 1 in Columbus). Except in Chicago, Model 4 had the largest magnitude of bias.

**Figure 9:**
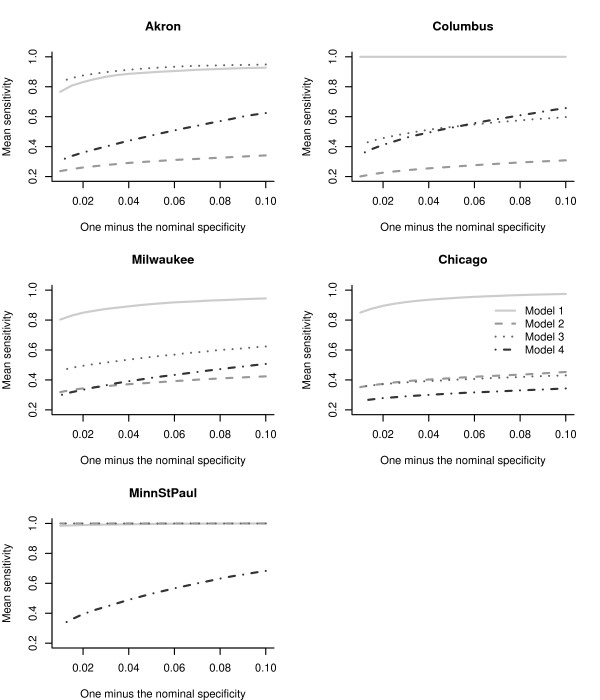
**Receiver operating characteristic curves for the four models**. Receiver operating characteristic curves (averaged over 500 simulations of an anthrax outbreak) for the detection of the anthrax outbreak signature in the chest radiographs at each of the five metropolitan children's hospitals using the four different models. The linear filter is used in each case.

Figure 9 compares the receiver operator characteristic (ROC) curves for the four models across the five EDs using the linear filter (Across all models, the sensitivity for the 1-day filter was the lowest while the 7-day and linear filters tended to have the highest sensitivity). To readily compare the models, we use one minus the nominal specificity on the x-axis. Except for Akron and Minneapolis/St. Paul, the random weekly and daily time series components in Model 1 yield higher sensitivities, compared to including a day-of-week covariate plus ARMA errors in Model 2, and day-of-week covariate plus no ARMA errors in Model 3. This is due to the insignificance of the day-of-week fixed effect in Models 2 and 3. In Akron, Model 3 has higher sensitivities than Model 1, suggesting that the time series errors are less relevant in outbreak signature detection for the test data. In Minneapolis/St. Paul Models 1, 2, and 3 have equivalent sensitivities. Across all cities either Model 2 or Model 4 had the lowest sensivities, indicating that these models are less reliable for outbreak signature detection.

In terms of sensitivity, except for Chicago, Model 3 tended to outperform Model 2 for all filters in the cities we studied. This is counter-intuitive, as we would expect that a model that includes the significant ARMA time series component (Model 2) should outperform one that did not contain any time series components (Model 3). In practice there is a tradeoff between estimation and prediction. Table 4 displays the Akaike information criterions (AICs) for the four models, fit to the training data of the five EDs. For each ED, a smaller AIC denotes a model that better fits the data. Model 3 performed better than Model 2 in one-step-ahead predictions (Figure 9), but Model 2 better explains the training data than Model 3 (Table 4). Using the AIC we find Model 1 fits best to the training data, even though it does not always yield the highest sensitivity.

**Table 4:** Akaike information criterions (AICs) for Models 1–4 fit to the training data for each ED

City	Model 1	Model 2	Model 3	Model 4
Akron	1600	1618	1790	1618
Columbus	1768	1803	1845	1852
Milwaukee	1645	1658	1664	1670
Chicago	1446	1474	1481	1475
Minn/St. Paul	1763	1777	1791	1915

## Discussion

Our intention in this study was to find a flexible set of statistical models that could be applied across a number of emergency departments. We employ time series models that include covariates, such as patient visit counts and ambient temperature, as well as random seasonal terms. We use chest-radiograph ordering data from emergency departments of five regional Midwest children's hospitals to detect signatures of respiratory outbreaks. We include visit counts series as a covariate in the chest radiograph  model to account for variations due to, for example, ED sizes, changes  of staff within the ED, and even some seasonalities across the time  period of interest. We use the temperature series as a surrogate measure of the influenza season – the colder months in the western hemisphere. This is a more accurate measure of the influenza season than using a fixed covariate such as a sinusoid. To reflect uncertainty in the variation of the influenza background over seasons, these models allow for randomness in the seasonal components. The use of random seasonal components is an advantage over traditional fixed effect models, since temporal patterns are not assumed to repeat precisely the same way. Thus, signature detection capabilities are improved for the majority of EDs – sensitivity is higher. For increased accuracy and timeliness, the use of our model for the data analysis should represent one component of an integrated detection system. Once a signal is triggered by any of our models, we recommend the use of clinical follow-up to corroborate or refute the emergence of a bona fide epidemic. For example, radiographs and medical charts will need to be reviewed to identify highly anomalous findings or groupings of aberrant findings that would be expected to be present at early stages of outbreaks. We believe that the approach utilized in this work will aid in this process and is more appropriate than models using fixed periodicities that do not have the ability to capture the underlying variabilities across seasons.

Of note, our study shows that there are similarities in the chest radiographs series from different EDs that can, for the most part, be modeled by similar time series models. Similarities of the time series model across EDs have a number of ramifications for detection of outbreak signatures. First, by borrowing information across the different EDs we can build more complicated multivariate time series models, possibly involving the joint modeling of chest radiograph and visit counts across locations. Second, we could use these models to jointly detect outbreak signatures across large spatial regions. In this context, Diggle et al. [20] use spatio-temporal Cox models to identify anomalies (real and artificial outbreaks) in the space-time distribution of gastrointestinal infections. But, some caution is needed because one potential drawback of aggregating data spatially is that the chance of detection can be reduced (data from unaffected areas will mask the outbreak signature, and increase the detection time) [21]. For localized outbreaks, there is still some utility in building models that borrow strength across EDs, even if the joint detection of outbreak signatures is not meaningful. In terms of these localized outbreaks, a geographically close site could act as a benchmark to judge detections at other sites. For example, under certain circumstances an epidemic detection signal triggered in Columbus, but not in Akron, could imply that some unusual event had occurred in Columbus. It should be noted, however, that even though there are similarities in the time series models, our simulations demonstrate that the sensitivity and timeliness to detect outbreak signatures using chest radiograph counts were different across EDs. These differences may limit the utility of our models in comparing signals across different EDs.

Our study has a number of limitations. We assume that the resulting series {*X*_*k*,*t*_}, after accounting for these covariates, is stationary. We also ignore the fact that an individual may have multiple ED visits. Furthermore, these data do not contain any known anthrax outbreaks. Instead, outbreak patterns are simulated using a simple stochastic model. Although more care needs to be taken when summarizing simulation results, we agree with Buckeridge, et al. [16] and Brookmeyer, et al. [17] that stochastic outbreaks are more realistic than deterministic ones. Other results (not presented here) indicate that the results are different (and possibly misleading) with a smooth deterministic outbreak rather than a stochastic one. We utilize the four filters used by Reis, et al. [2]. Although filter design is an active area of research, especially in engineering, we decided to only study these four simple and fairly easy to understand filters. In the future we could extend our analysis to different choices of filter. We view wavelet-based biosurveillance methods [1,8] as extensions of this idea to different linear time-invariant filters. An important issue in filter-based-prediction methods is the choice of covariates and the correlation structure for the time series model. Clearly, the properties of the time series model could change with time. One way to improve the prediction of outbreak signatures is to use time-varying models, with parameters that slowly evolve over time. The use of time-varying parameter models is analogous to a periodic review of the parameter values. Window-based estimation methods could be used to re-estimate the model parameters. In this case, the choice of window length is critical as it represents a compromise between the efficiency to estimate the model parameters, and the sensitivity and specificity of detection. Other issues to be taken into account include negative singularities (as defined by [8]) that can cause a drop in the sensitivity. Abnormal points (like holidays or severe weather) can cause "false alarms". Our solution to this problem is to jointly model chest radiograph and visit counts. So far, we have assumed that the visit counts are observed without error. Related to these issues, we are investigating methods that can be used to incorporate the uncertainty in parameter estimation upon the detection procedure.

## Conclusion

We present in this paper a stochastic model of chest radiograph ordering patterns and temperature as an adjunct to a biosurveillance system for detecting emerging respiratory-related epidemics, focusing on a potentially high-impact public health hazard such as inhalational anthrax. We show that in time series analysis of respiratory-related data it is important to capture important seasonal effects that are present in such data, as well as to consider the influence of important covariates that can be easily obtained and incorporated into the models. We also demonstrate the importance in assessing the sensitivity and specificity of these methods, of utilizing more realistic stochastic, rather than deterministic, models for outbreaks patterns. We demonstrate spatial homogeneity in chest radiograph data across EDs and suggest ways in which these observations may be used to improve regional biosurveillance for (re)emergent infections.

Regardless of the choice of filter, our simulations demonstrate that the specificity calculated using the training data varied across the five EDs studied (Figure 6). The tradeoff between specificity and sensitivity also varied by the ED (Figures 6 and 7). The 1-day filter, while closely matching the nominal specificity, performed poorly in terms of maximizing the sensitivity to detect an outbreak signature. The 7-day filter, by smoothing over the outbreak signature (Figure 5), maximized the sensitivity, but at a compromise to the actual specificity obtained (Figures 6 and 7). The linear and exponential filters provided a balance between the specificity and sensitivity. Detection using our covariate-based seasonal time series model performed well across all EDs compared with fixed-effects regression models or time series models that omitted seasonal terms and/or covariates (Figures 8 and 9).

## Competing interests

The author(s) declare that they have no competing interests.

## Authors' contributions

Dr. Bonsu provided the motivation, scientific background, and data for this work. Under supervision, N. Kim developed the preliminary simulation model for an anthrax attack. Dr. Craigmile worked on the filtering theory used in the paper, and together with N. Kim and Dr. Fernandez analyzed the data, planned and carried out the simulations, and wrote the paper. Dr. Craigmile took the lead with writing. All authors jointly edited this work.

## Appendix

### The SARIMA model

In city *k*, we define a SARIMA (*p*_*k*_, 0, *q*_*k*_) × (*P*_*k*_, 0, *Q*_*k*_) with period of seasonality *s *for the time series {*X*_*k*,*t*_} using the notation of Section 6.5 of Brockwell and Davis [19]. Letting *B *denote the backshift operator defined by *B*^*r*^*Z*_*k*,*t *_= *B*^*r*-1 ^*Z*_*k,t*-1 _for *r *> 0, with *B*^0 ^*Z*_*k*,*t *_= *Z*_*k*,*t*_, we define *X*_*k*,*t *_by

*φ*_*k*_(*B*)Φ_*k*_(*B*^*s*^)*X*_*k*,*t *_= *θ*_*k*_(*B*)Θ_*k*_(*B*^*s*^)*Z*_*k*,*t*_.

In this model the parameter *s *denotes the period of the seasonality. The characteristic polynomials for the SARIMA model are

φk(z)=1−φk,1z−…−φk,pkzpk;Φk(z)=1−Φk,1z−…−Φk,PKzPK;θk(z)=1+θk,1z+…+θk,qKzqK;Θk(z)=1+Θk,1z+…+Θk,QKzQK.
 MathType@MTEF@5@5@+=feaafiart1ev1aaatCvAUfKttLearuWrP9MDH5MBPbIqV92AaeXatLxBI9gBaebbnrfifHhDYfgasaacH8akY=wiFfYdH8Gipec8Eeeu0xXdbba9frFj0=OqFfea0dXdd9vqai=hGuQ8kuc9pgc9s8qqaq=dirpe0xb9q8qiLsFr0=vr0=vr0dc8meaabaqaciaacaGaaeqabaqabeGadaaakeaafaqadeabdaaaaeaaiiGacqWFgpGzdaWgaaWcbaGaem4AaSgabeaakiabcIcaOiabdQha6jabcMcaPaqaaiabg2da9aqaaiabigdaXiabgkHiTiab=z8aMnaaBaaaleaacqWGRbWAcqGGSaalcqaIXaqmaeqaaOGaemOEaONaeyOeI0IaeSOjGSKaeyOeI0Iae8NXdy2aaSbaaSqaaiabdUgaRjabcYcaSiabdchaWnaaBaaameaacqWGRbWAaeqaaaWcbeaakiabdQha6naaCaaaleqabaGaemiCaa3aaSbaaWqaaiabdUgaRbqabaaaaOGaei4oaSdabaGaeuOPdy0aaSbaaSqaaiabdUgaRbqabaGccqGGOaakcqWG6bGEcqGGPaqkaeaacqGH9aqpaeaacqaIXaqmcqGHsislcqqHMoGrdaWgaaWcbaGaem4AaSMaeiilaWIaeGymaedabeaakiabdQha6jabgkHiTiablAciljabgkHiTiabfA6agnaaBaaaleaacqWGRbWAcqGGSaalcqWGqbaudaWgaaadbaGaem4saSeabeaaaSqabaGccqWG6bGEdaahaaWcbeqaaiabdcfaqnaaBaaameaacqWGlbWsaeqaaaaakiabcUda7aqaaiab=H7aXnaaBaaaleaacqWGRbWAaeqaaOGaeiikaGIaemOEaONaeiykaKcabaGaeyypa0dabaGaeGymaeJaey4kaSIae8hUde3aaSbaaSqaaiabdUgaRjabcYcaSiabigdaXaqabaGccqWG6bGEcqGHRaWkcqWIMaYscqGHRaWkcqWF4oqCdaWgaaWcbaGaem4AaSMaeiilaWIaemyCae3aaSbaaWqaaiabdUealbqabaaaleqaaOGaemOEaO3aaWbaaSqabeaacqWGXbqCdaWgaaadbaGaem4saSeabeaaaaGccqGG7aWoaeaacqqHyoqudaWgaaWcbaGaem4AaSgabeaakiabcIcaOiabdQha6jabcMcaPaqaaiabg2da9aqaaiabigdaXiabgUcaRiabfI5arnaaBaaaleaacqWGRbWAcqGGSaalcqaIXaqmaeqaaOGaemOEaONaey4kaSIaeSOjGSKaey4kaSIaeuiMde1aaSbaaSqaaiabdUgaRjabcYcaSiabdgfarnaaBaaameaacqWGlbWsaeqaaaWcbeaakiabdQha6naaCaaaleqabaGaemyuae1aaSbaaWqaaiabdUealbqabaaaaOGaeiOla4caaaaa@A787@

The terms *φ*_*k*_(*z*) and *θ*_*k*_(*z*) define an autoregressive moving average process on the unit time scale, whereas the terms Φ_*k*_(*z*) and Θ_*k*_(*z*) define an autoregressive moving average process on a time scale of *s *units. Thus we can model dependencies simultaneously on two different time scales. It is customary to restrict to the class of *causal *time series models (e.g., [[19], Chapter 2]). The SARIMA model is causal (i.e., can be represented in terms of a moving average (MA) process of only past events) if *φ*_*k*_(*z*) ≠ 0 and Φ_*k*_(*z*) ≠ 0, for complex valued *z *such that |*z*| ≤ 1.

To complete the model we assume that {*Z*_*k*,*t*_} is a mean zero white noise process with innovation variance σk2
 MathType@MTEF@5@5@+=feaafiart1ev1aaatCvAUfKttLearuWrP9MDH5MBPbIqV92AaeXatLxBI9gBaebbnrfifHhDYfgasaacH8akY=wiFfYdH8Gipec8Eeeu0xXdbba9frFj0=OqFfea0dXdd9vqai=hGuQ8kuc9pgc9s8qqaq=dirpe0xb9q8qiLsFr0=vr0=vr0dc8meaabaqaciaacaGaaeqabaqabeGadaaakeaaiiGacqWFdpWCdaqhaaWcbaGaem4AaSgabaGaeGOmaidaaaaa@30F4@.

### Filtering

A discrete-time filter is a set of coefficients, {*f*_*l *_: *l *= ..., -2, -1, 0, 1, 2, ...}, where ∑_*l*_|*f*_*l*_| < ∞. The new time series {*Y*_*t*_} obtained by filtering the time series {*X*_*t*_} using {*f*_*l*_} is defined by the convolution

Yt=∑l=−∞∞flXt−l,
 MathType@MTEF@5@5@+=feaafiart1ev1aaatCvAUfKttLearuWrP9MDH5MBPbIqV92AaeXatLxBI9gBaebbnrfifHhDYfgasaacH8akY=wiFfYdH8Gipec8Eeeu0xXdbba9frFj0=OqFfea0dXdd9vqai=hGuQ8kuc9pgc9s8qqaq=dirpe0xb9q8qiLsFr0=vr0=vr0dc8meaabaqaciaacaGaaeqabaqabeGadaaakeaacqWGzbqwdaWgaaWcbaGaemiDaqhabeaakiabg2da9maaqahabaGaemOzay2aaSbaaSqaaiabdYgaSbqabaGccqWGybawdaWgaaWcbaGaemiDaqNaeyOeI0IaemiBaWgabeaaaeaacqWGSbaBcqGH9aqpcqGHsislcqGHEisPaeaacqGHEisPa0GaeyyeIuoakiabcYcaSaaa@41FB@

for each *t*. The actual operation that a filter carries out upon a time series depends on the values of the filter coefficients. Simple examples of a filter include the differencing filter defined by

fl={1l=0;−1l=1;0otherwise,
 MathType@MTEF@5@5@+=feaafiart1ev1aaatCvAUfKttLearuWrP9MDH5MBPbIqV92AaeXatLxBI9gBaebbnrfifHhDYfgasaacH8akY=wiFfYdH8Gipec8Eeeu0xXdbba9frFj0=OqFfea0dXdd9vqai=hGuQ8kuc9pgc9s8qqaq=dirpe0xb9q8qiLsFr0=vr0=vr0dc8meaabaqaciaacaGaaeqabaqabeGadaaakeaacqWGMbGzdaWgaaWcbaGaemiBaWgabeaakiabg2da9maaceqabaqbaeaabmGaaaqaaiabigdaXaqaaiabdYgaSjabg2da9iabicdaWiabcUda7aqaaiabgkHiTiabigdaXaqaaiabdYgaSjabg2da9iabigdaXiabcUda7aqaaiabicdaWaqaaiabb+gaVjabbsha0jabbIgaOjabbwgaLjabbkhaYjabbEha3jabbMgaPjabbohaZjabbwgaLjabcYcaSaaaaiaawUhaaaaa@4B81@

which corresponds to *Y*_*t *_= *X*_*t *_- *X*_*t*-1 _for each *t*, and the simple moving average filter of length 2*M *+ 1 whose filter is defined by

fl={1/(2M+1)l=−M,...,M;0otherwise.
 MathType@MTEF@5@5@+=feaafiart1ev1aaatCvAUfKttLearuWrP9MDH5MBPbIqV92AaeXatLxBI9gBaebbnrfifHhDYfgasaacH8akY=wiFfYdH8Gipec8Eeeu0xXdbba9frFj0=OqFfea0dXdd9vqai=hGuQ8kuc9pgc9s8qqaq=dirpe0xb9q8qiLsFr0=vr0=vr0dc8meaabaqaciaacaGaaeqabaqabeGadaaakeaacqWGMbGzdaWgaaWcbaGaemiBaWgabeaakiabg2da9maaceqabaqbaeaabiGaaaqaaiabigdaXiabc+caViabcIcaOiabikdaYiabd2eanjabgUcaRiabigdaXiabcMcaPaqaaiabdYgaSjabg2da9iabgkHiTiabd2eanjabcYcaSiabc6caUiabc6caUiabc6caUiabcYcaSiabd2eanjabcUda7aqaaiabicdaWaqaaiabb+gaVjabbsha0jabbIgaOjabbwgaLjabbkhaYjabbEha3jabbMgaPjabbohaZjabbwgaLjabc6caUaaaaiaawUhaaaaa@5280@

Thus, Yt=(2M+1)−1∑l=−MMXt+l
 MathType@MTEF@5@5@+=feaafiart1ev1aaatCvAUfKttLearuWrP9MDH5MBPbIqV92AaeXatLxBI9gBaebbnrfifHhDYfgasaacH8akY=wiFfYdH8Gipec8Eeeu0xXdbba9frFj0=OqFfea0dXdd9vqai=hGuQ8kuc9pgc9s8qqaq=dirpe0xb9q8qiLsFr0=vr0=vr0dc8meaabaqaciaacaGaaeqabaqabeGadaaakeaacqWGzbqwdaWgaaWcbaGaemiDaqhabeaakiabg2da9iabcIcaOiabikdaYiabd2eanjabgUcaRiabigdaXiabcMcaPmaaCaaaleqabaGaeyOeI0IaeGymaedaaOWaaabmaeaacqWGybawdaWgaaWcbaGaemiDaqNaey4kaSIaemiBaWgabeaaaeaacqWGSbaBcqGH9aqpcqGHsislcqWGnbqtaeaacqWGnbqta0GaeyyeIuoaaaa@44EB@, for each *t*.

Suppose that the radiograph series {*R*_*k*,*t*_} follows a time series model given by (1). Let *γ*_*X,k*_(·) denote the autocovariance function of the stationary error component {*X*_*k*,*t*_}. Consider only the last *m *values of the process *R*_*k*,*t*_, {*R*_*k,t*-1_, ..., *R*_*k,t*-*m*_}. Given the model parameters, by linearity of the prediction operator, the best linear one-step predictor of *R*_*k*,*t *_is given by

R^k,t=β0,t+βV,kVk,t+βT,kTk,t+X^k,t
 MathType@MTEF@5@5@+=feaafiart1ev1aaatCvAUfKttLearuWrP9MDH5MBPbIqV92AaeXatLxBI9gBaebbnrfifHhDYfgasaacH8akY=wiFfYdH8Gipec8Eeeu0xXdbba9frFj0=OqFfea0dXdd9vqai=hGuQ8kuc9pgc9s8qqaq=dirpe0xb9q8qiLsFr0=vr0=vr0dc8meaabaqaciaacaGaaeqabaqabeGadaaakeaacuWGsbGugaqcamaaBaaaleaacqWGRbWAcqGGSaalcqWG0baDaeqaaOGaeyypa0dcciGae8NSdi2aaSbaaSqaaiabicdaWiabcYcaSiabdsha0bqabaGccqGHRaWkcqWFYoGydaWgaaWcbaGaemOvayLaeiilaWIaem4AaSgabeaakiabdAfawnaaBaaaleaacqWGRbWAcqGGSaalcqWG0baDaeqaaOGaey4kaSIae8NSdi2aaSbaaSqaaiabdsfaujabcYcaSiabdUgaRbqabaGccqWGubavdaWgaaWcbaGaem4AaSMaeiilaWIaemiDaqhabeaakiabgUcaRiqbdIfayzaajaWaaSbaaSqaaiabdUgaRjabcYcaSiabdsha0bqabaaaaa@5477@

where

X^k,t=∑j=1mbk,jXk,t−j,
 MathType@MTEF@5@5@+=feaafiart1ev1aaatCvAUfKttLearuWrP9MDH5MBPbIqV92AaeXatLxBI9gBaebbnrfifHhDYfgasaacH8akY=wiFfYdH8Gipec8Eeeu0xXdbba9frFj0=OqFfea0dXdd9vqai=hGuQ8kuc9pgc9s8qqaq=dirpe0xb9q8qiLsFr0=vr0=vr0dc8meaabaqaciaacaGaaeqabaqabeGadaaakeaacuWGybawgaqcamaaBaaaleaacqWGRbWAcqGGSaalcqWG0baDaeqaaOGaeyypa0ZaaabCaeaacqWGIbGydaWgaaWcbaGaem4AaSMaeiilaWIaemOAaOgabeaakiabdIfaynaaBaaaleaacqWGRbWAcqGGSaalcqWG0baDcqGHsislcqWGQbGAaeqaaaqaaiabdQgaQjabg2da9iabigdaXaqaaiabd2gaTbqdcqGHris5aOGaeiilaWcaaa@4736@

denotes the one-step ahead prediction for the SARIMA process and {*b*_*k*,1_, ..., *b*_*k*,*m*_} are obtained from the solution of the set of *m *linear equations:

γX,k(h)=∑j=1mbk,jγX,k(h−j),h=1,...,m.
 MathType@MTEF@5@5@+=feaafiart1ev1aaatCvAUfKttLearuWrP9MDH5MBPbIqV92AaeXatLxBI9gBaebbnrfifHhDYfgasaacH8akY=wiFfYdH8Gipec8Eeeu0xXdbba9frFj0=OqFfea0dXdd9vqai=hGuQ8kuc9pgc9s8qqaq=dirpe0xb9q8qiLsFr0=vr0=vr0dc8meaabaqaciaacaGaaeqabaqabeGadaaakeaafaqabeqacaaabaacciGae83SdC2aaSbaaSqaaiabdIfayjabcYcaSiabdUgaRbqabaGccqGGOaakcqWGObaAcqGGPaqkcqGH9aqpdaaeWbqaaiabdkgaInaaBaaaleaacqWGRbWAcqGGSaalcqWGQbGAaeqaaOGae83SdC2aaSbaaSqaaiabdIfayjabcYcaSiabdUgaRbqabaGccqGGOaakcqWGObaAcqGHsislcqWGQbGAcqGGPaqkaSqaaiabdQgaQjabg2da9iabigdaXaqaaiabd2gaTbqdcqGHris5aOGaeiilaWcabaGaemiAaGMaeyypa0JaeGymaeJaeiilaWIaeiOla4IaeiOla4IaeiOla4IaeiilaWIaemyBa0gaaiabc6caUaaa@57CE@

The one-step prediction error process, {*E*_*k*,*t*_} is

Ek,t=Rk,t−R^k,t=Xk,t−X^k,t=Xk,t−∑j=1mbk,jXk,t−j=∑j=0mck,jXk,t−j,
 MathType@MTEF@5@5@+=feaafiart1ev1aaatCvAUfKttLearuWrP9MDH5MBPbIqV92AaeXatLxBI9gBaebbnrfifHhDYfgasaacH8akY=wiFfYdH8Gipec8Eeeu0xXdbba9frFj0=OqFfea0dXdd9vqai=hGuQ8kuc9pgc9s8qqaq=dirpe0xb9q8qiLsFr0=vr0=vr0dc8meaabaqaciaacaGaaeqabaqabeGadaaakeaacqWGfbqrdaWgaaWcbaGaem4AaSMaeiilaWIaemiDaqhabeaakiabg2da9iabdkfasnaaBaaaleaacqWGRbWAcqGGSaalcqWG0baDaeqaaOGaeyOeI0IafmOuaiLbaKaadaWgaaWcbaGaem4AaSMaeiilaWIaemiDaqhabeaakiabg2da9iabdIfaynaaBaaaleaacqWGRbWAcqGGSaalcqWG0baDaeqaaOGaeyOeI0IafmiwaGLbaKaadaWgaaWcbaGaem4AaSMaeiilaWIaemiDaqhabeaakiabg2da9iabdIfaynaaBaaaleaacqWGRbWAcqGGSaalcqWG0baDaeqaaOGaeyOeI0YaaabCaeaacqWGIbGydaWgaaWcbaGaem4AaSMaeiilaWIaemOAaOgabeaakiabdIfaynaaBaaaleaacqWGRbWAcqGGSaalcqWG0baDcqGHsislcqWGQbGAaeqaaOGaeyypa0ZaaabCaeaacqWGJbWydaWgaaWcbaGaem4AaSMaeiilaWIaemOAaOgabeaakiabdIfaynaaBaaaleaacqWGRbWAcqGGSaalcqWG0baDcqGHsislcqWGQbGAaeqaaaqaaiabdQgaQjabg2da9iabicdaWaqaaiabd2gaTbqdcqGHris5aaWcbaGaemOAaOMaeyypa0JaeGymaedabaGaemyBa0ganiabggHiLdGccqGGSaalaaa@79FC@

where we define *c*_*k*,0 _= 1 and *c*_*k*,*j *_= -*b*_*k*,*j *_for *j *= 1, ..., *m*. The process {*E*_*k*,*t*_} is a filtering of {*X*_*k*,*t*_} and so if {*X*_*k*,*t*_} is stationary, by the linear time invariant filtering result for stationary processes, {*E*_*k*,*t*_} is also stationary ([18], Theorem 4.10.1). Moreover for large enough *m*, if {*X*_*k*,*t*_} is an invertible time series process then we can approximate {*X*_*k*,*t*_} by an autoregressive, AR(*m*) process ([18], Theorem 4.4.3). Using this approximation we can argue that {*E*_*k*,*t*_} is approximately a mean zero Gaussian independent and identically distributed process with variance gamma *γ*_*X*,*k*_(0).

Let {*a*_0_, ..., *a*_*L*-1_} denote a pre-specified filter of width *L*. We define the detection process {*D*_*k*,*t*_} by filtering the error process {*E*_*k*,*t*_} using this filter. Thus,

Dk,t=∑h=0L−1ahEk,t−h.
 MathType@MTEF@5@5@+=feaafiart1ev1aaatCvAUfKttLearuWrP9MDH5MBPbIqV92AaeXatLxBI9gBaebbnrfifHhDYfgasaacH8akY=wiFfYdH8Gipec8Eeeu0xXdbba9frFj0=OqFfea0dXdd9vqai=hGuQ8kuc9pgc9s8qqaq=dirpe0xb9q8qiLsFr0=vr0=vr0dc8meaabaqaciaacaGaaeqabaqabeGadaaakeaacqWGebardaWgaaWcbaGaem4AaSMaeiilaWIaemiDaqhabeaakiabg2da9maaqahabaGaemyyae2aaSbaaSqaaiabdIgaObqabaGccqWGfbqrdaWgaaWcbaGaem4AaSMaeiilaWIaemiDaqNaeyOeI0IaemiAaGgabeaaaeaacqWGObaAcqGH9aqpcqaIWaamaeaacqWGmbatcqGHsislcqaIXaqma0GaeyyeIuoakiabc6caUaaa@4628@

By the filtering result for stationary processes, stationarity of {*X*_*k*,*t*_} implies that {*D*_*k*,*t*_} is also a stationary process. For large enough *m*, since {*E*_*k*,*t*_} is approximately a white noise process,{*D*_*k*,*t*_} is approximately a moving average process of order *L *- 1 with coefficients *θ*_0 _= 1, *θ*_*j *_= *a*_*j*-1_/*a*_0 _for *j *= 1, ..., *L *- 1, and innovation variance a02γX,k(0)
 MathType@MTEF@5@5@+=feaafiart1ev1aaatCvAUfKttLearuWrP9MDH5MBPbIqV92AaeXatLxBI9gBaebbnrfifHhDYfgasaacH8akY=wiFfYdH8Gipec8Eeeu0xXdbba9frFj0=OqFfea0dXdd9vqai=hGuQ8kuc9pgc9s8qqaq=dirpe0xb9q8qiLsFr0=vr0=vr0dc8meaabaqaciaacaGaaeqabaqabeGadaaakeaacqWGHbqydaqhaaWcbaGaeGimaadabaGaeGOmaidaaGGacOGae83SdC2aaSbaaSqaaiabdIfayjabcYcaSiabdUgaRbqabaGccqGGOaakcqaIWaamcqGGPaqkaaa@380A@.

We declare evidence of an outbreak signature (test positive), at time *t *if the observed detection value *D*_*k*,*t *_is larger than a given threshold. For a fixed value of *α*, let 1 - *α *denote the specificity (the probability of a true-negative). Similarly, the sensitivity is defined to be the probability of true positive. The threshold, *τ*_*k*,*α*_, is chosen by solving P (*D*_*k*,*t *_> *τ*_*k*,*α*_) = *α *for *τ*_*k*,*α*_. Either we can estimate this value from the data using the 1 - *α *quantile of the {*D*_*k*,*t*_} process of non-outbreak-based training data, or if {*X*_*k*,*t*_} is Gaussian then for large *m*,

P(Dk,t>τk,α)≈P(Z>τk,αγX,k(0)∑h=0L−1ah2)=α,
 MathType@MTEF@5@5@+=feaafiart1ev1aaatCvAUfKttLearuWrP9MDH5MBPbIqV92AaeXatLxBI9gBaebbnrfifHhDYfgasaacH8akY=wiFfYdH8Gipec8Eeeu0xXdbba9frFj0=OqFfea0dXdd9vqai=hGuQ8kuc9pgc9s8qqaq=dirpe0xb9q8qiLsFr0=vr0=vr0dc8meaabaqaciaacaGaaeqabaqabeGadaaakeaacqWGqbaucqGGOaakcqWGebardaWgaaWcbaGaem4AaSMaeiilaWIaemiDaqhabeaakiabg6da+GGaciab=r8a0naaBaaaleaacqWGRbWAcqGGSaalcqWFXoqyaeqaaOGaeiykaKIaeyisISRaemiuaa1aaeWaaeaacqWGAbGwcqGH+aGpdaWcaaqaaiab=r8a0naaBaaaleaacqWGRbWAcqGGSaalcqWFXoqyaeqaaaGcbaWaaOaaaeaacqWFZoWzdaWgaaWcbaGaemiwaGLaeiilaWIaem4AaSgabeaakiabcIcaOiabicdaWiabcMcaPmaaqadabaGaemyyae2aa0baaSqaaiabdIgaObqaaiabikdaYaaaaeaacqWGObaAcqGH9aqpcqaIWaamaeaacqWGmbatcqGHsislcqaIXaqma0GaeyyeIuoaaSqabaaaaaGccaGLOaGaayzkaaGaeyypa0Jae8xSdeMaeiilaWcaaa@5F82@

where *Z *is a standard normal random variable. The solution for the threshold under this approximation is

τk,α=(γX,k(0)∑h=0L−1ah2)1/2Φ−1(1−α),
 MathType@MTEF@5@5@+=feaafiart1ev1aaatCvAUfKttLearuWrP9MDH5MBPbIqV92AaeXatLxBI9gBaebbnrfifHhDYfgasaacH8akY=wiFfYdH8Gipec8Eeeu0xXdbba9frFj0=OqFfea0dXdd9vqai=hGuQ8kuc9pgc9s8qqaq=dirpe0xb9q8qiLsFr0=vr0=vr0dc8meaabaqaciaacaGaaeqabaqabeGadaaakeaaiiGacqWFepaDdaWgaaWcbaGaem4AaSMaeiilaWIae8xSdegabeaakiabg2da9maabmaabaGae83SdC2aaSbaaSqaaiabdIfayjabcYcaSiabdUgaRbqabaGccqGGOaakcqaIWaamcqGGPaqkdaaeWbqaaiabdggaHnaaDaaaleaacqWGObaAaeaacqaIYaGmaaaabaGaemiAaGMaeyypa0JaeGimaadabaGaemitaWKaeyOeI0IaeGymaedaniabggHiLdaakiaawIcacaGLPaaadaahaaWcbeqaaiabigdaXiabc+caViabikdaYaaakiabfA6agnaaCaaaleqabaGaeyOeI0IaeGymaedaaOGaeiikaGIaeGymaeJaeyOeI0Iae8xSdeMaeiykaKIaeiilaWcaaa@55EB@

where Φ^-1^(·) denotes the inverse cumulative distribution function for a standard normal random variable.

The filter-based detection method requires knowledge of the "true" values of the model parameters. In our work, we replace the parameters with their maximum likelihood estimates.

## Pre-publication history

The pre-publication history for this paper can be accessed here:


